# The Risk of Polychlorinated Biphenyls Facilitating Tumors in Hawaiian Green Sea Turtles (*Chelonia mydas*)

**DOI:** 10.3390/ijerph15061243

**Published:** 2018-06-12

**Authors:** Muting Yan, Huayue Nie, Wenjing Wang, Yumei Huang, Qing X. Li, Jun Wang

**Affiliations:** 1 College of Marine Sciences, South China Agricultural University, Guangzhou 510642, China; marineymt@scau.edu.cn (M.Y.); hynhy@stu.scau.edu.cn (H.N.); wenjing1379@163.com (W.W.); huangyumei@scau.edu.cn (Y.H.); 2 Joint Laboratory of Guangdong Province and Hong Kong Region on Marine Bioresource Conservation and Exploitation, South China Agricultural University, Guangzhou 510642, China; 3 Department of Molecular Biosciences and Bioengineering, University of Hawaii at Manoa, Honolulu, HI 96822, USA; qingl@hawaii.edu

**Keywords:** Hawaiian green turtle, PCBs, fibropapillomatosis, tumor

## Abstract

The Hawaiian green turtle (*Chelonia mydas*) is on the list of threatened species protected under the U.S. Endangered Species Act in 1978 in large part due to a severe tumor-forming disease named fibropapillomatosis. Chemical pollution is a prime suspect threatening the survival of *C. mydas*. In this study, PCBs concentrations were determined in 43 *C. mydas* plasma samples archived on Tern Island. The total PCBs concentration in male *C. mydas* (mean 1.10 ng/mL) was two times more than that of females (mean 0.43 ng/mL). The relationship between straight carapace length and PCBs concentration in females has also been studied, which was negatively related. To figure out the possible existence of correlations between PCBs and tumor status, we measured the PCBs concentration in turtles with no tumor, moderate or severe tumor affliction. PCBs concentration of two afflicted groups was much higher than the healthy group, suggesting that PCBs may play a role in fibropapillomatosis in Hawaiian green turtle.

## 1. Introduction

The hawaiian green turtle is a threatened species mainly because of over exploitation for their shells in the past. Apart from over-harvesting, some human impacts have also contributed to their decline these years, including nesting site destruction, incidental capture in fishing gear, egg collection and environmental pollution [1]. Among the seven existing sea turtle species, the green turtle is the largest hard-shell species and adults commonly reach 100 cm on average in straight carapace length (SCL). As they grew, SCL increased almost evenly every year. Thus, SCL was often used as an indicator to estimate the age of *C. mydas* [2]. In Hawaii, over 90% of *C. mydas* were nesting at the French Frigate Shoals inside the U.S. National Wildlife Refuge. Tern Island, where the turtles were sampled in this study, was a tiny island lied in the French Frigate shoals, approximately 490 miles northwest of Oahu (166° W longitude, 28° N latitude) [3].

Fibropapillomatosis (FP) is a serious and prevalent disease threatening the survival of green turtles, likely caused by a novel herpesvirus named fibropapilloma-associated turtle herpesvirus [4]. The affected turtle may develop tumors around soft skin like the neck and chin, making it difficult for the turtle to eat and making it vulnerable to predators [5]. The growth of tumors disrupts organ functions and commonly leads to the death of turtles. As FP primarily affects highly reproductive adults and large juveniles, the threat to the long-term survival of this species has been posed for the last 50 years [6]. Initially, the infection rate of the captured turtles was only 1.5%, indicating that this disease was accidental at that time [7]. However, in the last two decades, FP has become prevalent in some populations, thus destroying the recovery of this threatened species [8,9]. In Hawaii, FP morbidity reached 75% of stranded green turtles captured at Kaneohe Bay and West Maui in 2010 [10]. Recently, researchers have found that the rise of FP is coincident with some environmental pollutant temporal trends, such as persistent organic pollutant (POP) [11].

Polychlorinated biphenyls (PCBs) are a kind of synthetic organic chloride compound that have been widely used in products as plasticizers, transformer dielectric fluids, pesticide additives and paints since the 1930s. Considering the serious threat to the environment and public health posed by PCBs, most countries restricted PCBs production and use during the 1980s [12,13,14]. As a persistent organic pollutant, PCBs are commonly amplified and transferred along the food chain especially in aquatic organisms, which could be dangerous and fatal even if in very low concentrations [15,16,17,18]. Furthermore, studies have revealed that most marine vertebrates such as birds, dolphins, turtles and fish all contained different concentrations of PCBs that far exceeded the circumstance content [15,19,20,21]. With the progress of environmental deterioration, toxicological effects of PCBs on aquatic animals have attracted more attention in recent years [22]. Several studies have suggested that PCBs can impair turtles’ survival and reproduction, as well as their immune system and organ functions [23,24,25]. In this study, plasma samples were used to determine PCBs concentration associated with different sex or age, and to evaluate the effects of PCBs on tumor aggravation in Hawaiian green turtles.

## 2. Materials and Methods

### 2.1. Sample Information

The samples were collected by the U.S. Fish and Wildlife Service on Tern Island during the nesting season. The sex of the turtles was determined based on the secondary sexual characteristics, such as length of the tail and nails. Both males and females were sampled for blood, relevant data (size measurements, weight) was obtained as well. A total of 43 turtles were sampled. The whole blood samples were stored at −20 °C until they were extracted.

### 2.2. Extraction and Cleanup

The whole blood samples collected from the turtles were extracted with a pressurized fluid extractor (Dionex, Sunnyvale, CA, USA). The whole blood was weighed and 1 mL of methanol was added for 2 g of wet blood to lyse the red blood cells. The samples were then allowed to sit overnight before extracting, and a large amount of solvent evaporates. The blood samples are mixed with sodium sulfate (1:3 ratio of sample:sodium sulfate). The 33 mL ASE extraction cell is first filled with 3.0 g of acidified alumina for the collection of fats from the extract. The sample mixed with sodium sulfate is placed on top of the acidified alumina, and the remaining volume is filled with clean Ottawa sand. The samples are extracted with hexane:acetone (1:1) at a pressure of 1500 psi and temperature of 150 °C with 2 static cycles of 20 min each.

The blood extracts are concentrated to 1 mL, and then subjected to column chromatographic cleanup. Cleanup procedure was performed as described in a previous study [26]. The extract was loaded onto the column, and eluted with 16 mL hexane:methylene chloride, 5:1 (*v*/*v*). The eluent was concentrated, and ready for GC-ECD analysis.

### 2.3. PCB Determination

A majority of the blood samples were analyzed on a Hewlett Packard 5890 gas chromatograph-electron capture detector (GC-ECD) (Hewlett-Packard, Avondale, PA, USA). The GC column used was a DB-5 capillary column (50 m × 0.20 mm i.d. × 0.11 μm). Helium was used as the carrier gas. The initial oven temperature was 120 °C and it was linearly ramped at 1 °C/min to 260 °C. The injector and detector were set to 270 °C and 320 °C, respectively. Concentrations of the PCBs were calculated from external PCB standards (Accustandard Inc., New Haven, CT, USA).

Some of the blood samples were also analyzed with a Varian GC-ECD/ion trap mass spectrometer Saturn 2000 (Walnut Creek, CA, USA). The column used was a ZB-1 capillary column, with 60 m × 0.20 mm i.d. × 0.25 μm film thickness (Phenomenex, CA, USA). High purity helium (grade 5.5) was used as the carrier gas. The initial oven temperature was 120 °C and it was linearly ramped at 1 °C/min to 260 °C, and the injector was set to 270 °C. The column eluent is split between the ECD detector and the ion trap mass spectrometer in a 1:10 ratio, respectively. The ECD data were used for quantitation of the individual PCB congeners, and external standards were used for determining the concentration (Accustandard Inc., New Haven, CT, USA).

The blood samples were analyzed two times on the same column, with varying initial oven times, ramping rate and final oven temperatures. The retention index was determined for both experimental conditions. The retention index for the PCBs should be similar, while the retention index for other compounds will differ. With this information, the PCBs can be distinguished from the other compounds in the sample.

### 2.4. Statistical Analysis

All numerical data were presented as the mean ± standard deviation (SD). The means of male and female were compared by Student’s *t*-test, as well as that of younger and older groups. The comparison of means in healthy, moderate affliction and severe affliction groups was conducted by one-way ANOVA analysis and Duncan’s multiple range test. The differences were significant at *p* < 0.05 in all cases.

## 3. Results and Discussion

### 3.1. PCBs Concentrations in Healthy C. mydas of Different Sexes

The PCBs concentrations in the plasma from green turtles of different sexes are listed in Table 1. Straight carapace length (SCL) was used as an indicator to estimate the age of *C. mydas* [2]. The SCLs of male and female were both ranged from 82 to 90 cm (mean 85.0 ± 2.03, 85.7 ± 2.44, respectively), indicating that these two groups maybe almost the same age. The total PCBs concentration in male *C. mydas* (mean 1.10 ng/mL) was two times more than that of females (mean 0.43 ng/mL). X.S. Miao et al. analyzed the total PCB in liver and adipose fat samples from three stranded green turtles on Oahu, which ranged from 45–58 ng/g in liver samples and 73–665 ng/g in adipose fat [27]. The significant lower PCB concentration in plasma could be related to the weaker accumulation and faster metabolism of blood. Similarly, Keller et al. measured blood PCB concentrations and observed a significant difference compared with PCB concentrations measured in other tissue [28]. In *C. mydas* and *Eretmochelys imbricata*, the PCB concentration was determined was and similar to our reports (0.53 ng/mL and 0.19 ng/mL, respectively) [1].

Actually, PCBs concentrations in females are expected to be lower, as has been reported in other turtles [29,30]. Several hypotheses may explain the lower PCB concentration in female green turtles. A possible reason for this pattern is maternal transfer. Indeed, females often transfer part of contamination to eggs in oviparous species, owing to the lipophilicity of organic compounds [31]. The maternal transfer of contaminants has been described in many marine vertebrates, including turtles, birds and mammals [32,33,34].

Besides, the lower PCBs concentration in females may be caused by them reducing their food intake. During the nesting season, females require massive energy to lay numerous eggs, which may go through the nesting season with little or no food intake and exhibit an obvious weight loss [35,36,37]. Contaminant in plasma could decrease along with the reduced food intake, as blood can reflect the recent absorption of contaminants in turtles [38]. Males spending more time on foraging areas may suffer a longer trophic contamination, resulting in higher concentration of PCBs.

### 3.2. Concentration of PCBs in Healthy Female C. mydas of Different Ages

According to the SCL, female turtles of different ages were divided into two groups. Table 2 shows the PCBs concentrations measured in the green turtles included the younger group (YG, *n* = 13) and the older group (OG, *n* = 11). The total PCBs concentrations are inversely related to the SCL. YG (mean 88.7 cm) contained higher total PCBs concentration (0.52 ng/mL), while OG (mean 94.1 cm) contained 0.15 ng/mL of PCBs, significantly lower than YG (*p* < 0.05). The result was consistent with a previous study that had showed that PCBs concentration decreased with increasing SCL in the Hawaiian green sea turtle, but without eliminating the effects of sex and age ratios in turtle samples [39].

We could attribute such a reduction to some possible reasons. First, the low PCBs concentration in older Hawaiian green sea turtle was likely related to diet. Actually, in other marine turtles, contaminant concentration has been confirmed to be closely connected to dietary preference [37,40,41]. In addition, organic pollutants enter sea turtles trough their diet, concentration of which in blood could be an indicator of their food intake [42]. As green sea turtles mature to adulthood, they change diet from eating sponges, seaweed, jellyfish and cuttlefish to sea grasses and algae, gradually becoming an herbivore [43]. As fish becomes a smaller part of the turtle’s diet, the amount of PCBs may slowly decrease during this transition. The bigger turtles are, the longer their herbivorous phase, undergoing a dilution of PCBs with age [44].

Secondly, a possible reason is maternal transfer, as mentioned above. Similarly, a significantly higher profile of PCBs contamination of juvenile loggerhead sea turtles than adults from Eastern Atlantic was observed by María Camacho et al., possibly because juvenile turtles were not at breeding age [45]. Large turtles may have been laying eggs for a longer time than smaller ones, off-loading more contaminant through maternal transfer. As the age and spawning frequency increased, PCBs in female turtles might be gradually released and decreased.

### 3.3. PCBs Concentration and Tumor Status in Hawaiian Green Turtles

**Figure 1:**
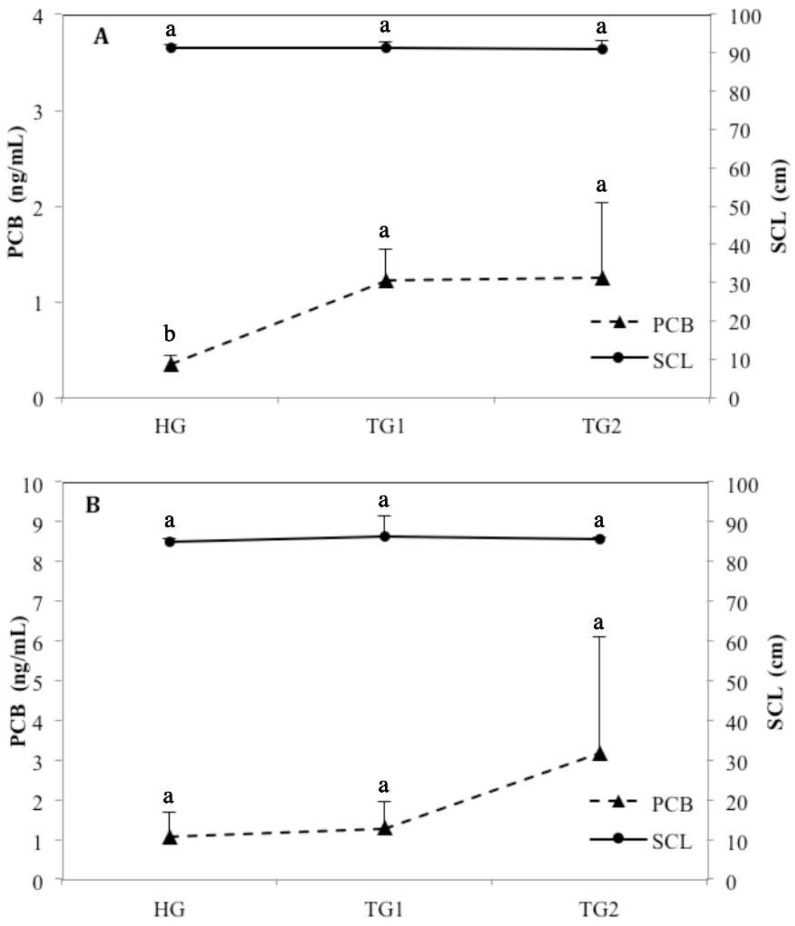
Tumor status and PCB concentration in the female (**A**), and male (**B**) green turtles collected from Hawaii. Data were presented as mean + standard error. Different lowercase letters indicate significant differences (*p* < 0.05; Duncan’s multiple range test). (HG: healthy group; TG1: moderate afflicted group; TG2: severe afflicted group).

To figure out the possible existence of correlations between PCBs contamination and tumor status, we measured the PCBs concentration in female turtles of different tumor status. According to the size and number of tumors, female turtles were divided into three groups, no tumor (HG, *n* = 24), moderate affliction (TG1, *n* = 7) or severe affliction (TG2, *n* = 2). Severely afflicted turtles showed the highest PCBs concentrations in the plasma (mean 1.25 ng/mL), followed by the moderately afflicted ones (mean 1.22 ng/mL). As described before, the PCBs concentration in healthy female turtles were much lower (mean 0.33 ng/mL). Given that there was no significant difference in SCL (*p* = 0.996), a significant increase was observed in PCB concentration along with tumor aggravation (*p* = 0.002) (Figure 1A). The close PCB concentration in TG1 and TG2 may be attributed to different exposure of limited samples to PCBs, resulting in a high degree of variation of concentrations of PCBs detected in TG2. In male turtles, PCBs concentration of severe afflicted turtles were much higher than the other two groups (Figure 1B). However, due to the limited samples (HG, *n* = 5; TG1, *n* = 3; TG2, *n* = 2) and large variance, no significant difference was observed in the male groups neither (*p* = 0.460). Notably, PCBs concentration of afflicted females and males were both higher than the healthy groups, suggesting that PCB may be associated with FP in Hawaiian green sea turtles.

Previous studies have demonstrated that PCB contamination could cause serious disease in other species. J. Orós et al. suggested that PCB in tissues was associated with the lesions in Atlantic turtle, including poor physical condition, cachexia and septicaemia [46]. Besides, chronic exposure to PCBs may alter immunity and the PCBs concentration was correlated with the degree of emaciation in red-eared sliders [47]. Recently, a Fish Health Assessment study has described that a range of PCB contamination in the Hudson River caused ovarian atresia in yellow perch [48]. In a previous study, X.S. Miao et al. had mentioned the FP in Hawaiian green sea turtle and determined the PCBs concentration in two male turtles with different tumor status. PCBs concentration of the severe afflicted one was 116 ng/g in adipose, while the moderate afflicted one was only 73.1 ng/g. However, due to the limited samples, authors were unable to analyze the relationship between PCB concentration and tumor status in the Hawaiian green sea turtle [27]. J. Keller et al. observed an increased FP score in Hawaiian green sea turtles with higher PCBs concentration. Unlike our studies, the turtles they investigated included adult and juvenile, mostly juvenile, without focusing on sexual distinction. Thus, considering the influence of multiple factors, the specific impact of PBCs in FP is difficult to define [39].

In our study, there was a trend of much higher PCBs concentration in afflicted turtles. The severe afflicted group had the highest concentration of PCBs, which might suggest that PCBs could contribute to the FP in adult green turtles. However, the mechanism of PCB causing tumors in Hawaiian green sea turtles needs further investigation.

### 3.4. Comparison of PCB Concentration in Blood with Other Turtles

The PCB concentrations analyzed here were compared to concentrations reported in other species of turtles collected in different locations (Table 3).

In general, PCBs contamination was detected in turtles whether from sea or freshwater with different degrees of concentration. PCBs concentration in Hawaiian green sea turtles was much lower than that in *Caretta caretta* from Canary Island and *Dermochelys coriacea* from French Guiana, but higher than that in loggerhead sea turtles from Cape Verde. The differences found in these species of turtles may due to the different degree of pollution in these areas. PCB concentration of loggerhead sea turtles from Cape Verde were quite low, as Cape Verde was an underdevelopment country with little industry or pollution [33]. Compared with Cape Verde, Hawaii is a prosperous region with highly developed industries and tourism. Hawaiian green sea turtles may be exposed to more pollutants during their migration along the coasts of the main Hawaiian Islands. Diamondback terrapin spans estuaries along the east coast of the US [51]. Frequent and intensive human activities resulted in high PCB pollution in turtles. Diet, sex, age, physical condition and the season of sampling may also be contributing factors for different PCB concentration in other turtles.

## 4. Conclusions

This study determined the concentrations of PCBs in male *C. mydas* and nesting females. The different concentrations between sexes were also examined, which was much higher in males than that in females. PCBs concentration was negatively related to the SCL in female turtles, indicating that PCBs were diluted as they grow, possibly due to the diet transition or maternal transfer. The relationship between PCBs in females and eggs was difficult to analyze due to a lack of paired samples. In addition, increased PCB concentration was observed in turtles with the aggravation of tumors, suggesting that PCBs may play a role in FP in the Hawaiian green sea turtle.

## Figures and Tables

**Table 1:** PCBs concentration in plasma samples from healthy green turtle collected on Tern Island, Hawaii.

Sex	SCL ^1^ (cm)		Weight (kg)	PCBs (ng/mL)		*p*-Value
	Mean (SD ^2^)	Range	Range	Mean (SD)	Range	
Male (*n* = 5)	85.0 (2.03)	82.6–88.2	83.0–88.9	1.10 (1.33)	0.04–3.11	<0.05
Female (*n* = 5)	85.7 (2.44)	82.6–89.1	88.5–113.4	0.43 (0.31)	0.07–0.83	

^1^ SCL = Straight carapace length; ^2^ SD = standard deviation.

**Table 2:** PCBs concentration in plasma samples of female Hawaiian green turtle in different ages.

Group	SCL ^3^ (cm)		Weight (kg)	PCBs (ng/mL)		*p*-Value
	Mean (SD ^4^)	Range	Range	Mean (SD)	Range	
YG ^1^ (*n* = 13)	88.7 (2.92)	82.6–92.0	78.9–125.2	0.52 (0.51)	0.10–1.92	<0.05
OG ^2^ (*n* = 11)	94.1 (2.02)	92.5–98.4	96.2–134.7	0.15 (0.11)	0.05–0.38	

^1^ YG = younger group; ^2^ OG = older group; ^3^ SCL = Straight carapace length; ^4^ SD = standard deviation.

**Table 3:** Average PCBs concentration in blood from other turtles.

Species	Locality	Sex	∑PCB	Reference
*Caretta caretta* ^b^	Cape Verde	Female	0.15 ^A^	[45]
*Caretta caretta* ^a^	Canary Islands	Female and male	3.77 ^A^	[45]
*Caretta caretta* ^a^	North Caroline	Female and Male	5.56 ^B^	[23]
*Malaclemys terrapin* ^b^	Barnegat Bay	Female	1050 ^B^	[49]
*Dermochelys coriacea* ^b^	French Guiana	Female	1.26 ^A^	[32]
*Chelydra serpentina* ^b^	Hudson River	Female	125 ^B^	[29]
*Chelydra serpentina* ^b^	Hudson River	Male	475 ^B^	[29]
*Chelydra serpentina* ^b^	Ashtabula River	Female	107.8 ^B^	[50]

^A^ Value expressed as ng/mL; ^B^ Value expressed as ng/g wet mass; ^a^ Young turtles; ^b^ Adult turtles.
